# Megadose Methyl-Prednisolone (MDMP) for Autoimmune Hemolytic Anemia

**DOI:** 10.4274/Tjh.2013.0052

**Published:** 2013-06-05

**Authors:** Şinasi Özsoylu

**Affiliations:** 1-1 Fatih University, School of Medicine, Department of Hematology, Ankara, Turkey

## TO THE EDITOR

Üsküdar Teke and colleagues originally used 1 mg kg^–1^ day^–1^ methylprednisolone (MDMP) for their patient with autoimmune hemolytic anemia without improvement, and after 3 weeks of steroid treatment splenic infarct developed. The patient was splenectomized but the hemolytic anemia again did not improve. Therefore, azathioprine was prescribed [1-1].

A 36-year-old woman with severe autoimmune hemolytic anemia was sent to me from the Netherlands, who was treated for more than 1.5 years with corticosteroid. Her anemia and hemolysis responded promptly to MDMP administration without disappearance of Coombs positivity [1-2].

The authors searched for causes of thrombophilia in their patient because of splenic infarct, without evaluation of the fibrinolytic system.

Since the coagulation depends on the dynamic balance between procoagulant and fibrinolytic activities, the later system should also be evaluated in the case of splenic infarct, I believe.

On this occasion, I would like to bring to attention that high homocysteine levels’ relation to thrombosis is doubtful, as was previously studied [1-3].
