# Supplementary material for: Megadose Methyl-Prednisolone (MDMP) for Autoimmune Hemolytic Anemia
Source: Turk J Haematol. 2013 Jun 5;30(2):228–9. doi: 10.4274/Tjh.2013.0052 (PMC3894616; doi:10.4274/Tjh.2013.0052)
Supplement: Related texts

# Reply

**Authors:** Hava Üsküdar Teke, Samet Karahan

**Affiliations:** 2-1 Eskisehir Osmangazi University, Faculty of Medicine, Department of Hematology, Eskisehir, Turkey; 2-2 Kayseri Education and Research Hospital, Department of Internal Medicine, Kayseri, Turkey

We want to thank for the comments of Şinasi ÖZSOYLU, MD. The new diagnosis in primary (idiopathic) autoimmune hemolytic anemia patients is suggested as glucocorticoids (steroids), prednison 1 mg/kg/day or metilprednisolon intravenously. With this dosage it is recommended to continue the treatment until the target hemoglobin 

value >10 g/dl and hematocrit value >30% are reached and if the target values are not reached within 3 weeks, second step of treatment should be applied [2-1]. In our case after 3 weeks steroid treatment, both symptomatic splenic infarct development and due to no result of 3 weeks steroid treatment splenectomy is applied simultaneously as a second step treatment. In our case due to splenic infarct development in accordance with etiology trombophilia investigation are observed [2-2]. As Dr. Özsoylu has already specified, due to the coagulation being dependant on the dynamic balance between procoagulant and fibrinolytic activity, fibrinolytic system could have been evaluated as well. However, because of the insufficient laboratory conditions, investigation about the fibrinolytic system couldn’t have been done.

## References

[2-1] Lechner K, Jager U (2010). How I treat autoimmune hemolytic anemias in adults. Blood.

[2-2] Teke HÜ, Karahan S, Gümüş Ü (2012). Splenic infarct in a patient with autoimmune hemolytic anemia. Turk J Hematol.
